# Beverage consumption in an Alaska Native village: a mixed-methods study of behaviour, attitudes and access

**DOI:** 10.3402/ijch.v75.29905

**Published:** 2016-02-24

**Authors:** Deena Elwan, Peter de Schweinitz, Janet M. Wojcicki

**Affiliations:** 1 Department of Pediatrics, University of California, San Francisco, San Francisco, CA, USA; 2 Chief Andrew Isaac Health Center, Tanana Chiefs Conference, Fairbanks, AK, USA

**Keywords:** sugar-sweetened beverages, water, Alaska Native, obesity, nutrition

## Abstract

**Background:**

American Indians/Alaska Natives (AI/AN) have the highest prevalence of obesity for any racial/ethnic group. Previous studies examining risk factors for obesity have identified excessive sugar-sweetened beverage (SSB) and inadequate water consumption as major risk factors for this population group. The historical scarcity of water in rural Alaska may explain consumption patterns including reliance on SSBs and other packaged drinks.

**Methods:**

Our study was designed to assess SSB, water and other beverage consumption and attitudes towards consumption in Alaska Native children and adults residing in rural Alaska. During summer 2014, 2 focus groups were conducted employing community members in a small rural village more than 200 air miles west of Fairbanks, Alaska. Interviews were completed with shop owners, Early Head Start and Head Start program instructors (n=7). SSB and total beverage intakes were measured using a modified version of the BEVQ-15, (n=69).

**Results:**

High rates of SSB consumption (defined as sweetened juice beverages, soda, sweet tea, energy drink or sports drinks) and low rates of water consumption were reported for all age groups in the village. All adolescents and 81% of children reported drinking SSBs at least once per week in the last month, and 48% of adolescents and 29% of younger children reported daily consumption. Fifty-two per cent of adults reported consuming SSBs at least once per week and 20% reported daily consumption. Twenty-five per cent of adolescents reported never drinking water in the past month, and 19% of younger children and 21% of adults did not consume water daily.

**Conclusion:**

Alaska Native children and adults living in the Interior Alaska consume high amounts of SSBs including energy drinks and insufficient amounts of water. Interventions targeting beverage consumption are urgently needed for the Alaska Native population in rural Alaska.

The prevalence of overweight and obesity in the United States has steadily increased since the 1970s (1,2) with the current prevalence of combined overweight and obesity (BMI≥25 for adults and BMI percentile ≥85% for children) equal to 22.8% for young children (aged 2–5), 34% for older children (aged 6–11), 34.5% for adolescents (aged 12–19) and 69% for adults (3). Previous studies show that overweight and obese children are more likely to become obese adults (4). On the national level, the prevalence of extreme obesity (BMI≥120% of the 95th percentile for children of the same sex and age) fell among every ethnic group except the American Indian/Alaska Native (AI/AN) population from 1998 to 2010 (5).

Nationally, the AI/AN population has the highest prevalence of obesity of any racial/ethnic group (6). According to a 2011 Indian Health Service (IHS) report, over 80% of AI/AN adults are overweight or obese (7). In Alaska Native adults, between 1991 and 2012, obesity increased from 16 to 35% (8). Alaska Native adolescents are also significantly more likely than white adolescents to be obese, with 16% of Alaska Native Alaskan high school students considered obese compared with 10% of white ones in Alaska (8). AI/AN preschoolers were almost twice as likely (31.2%) as their U.S. counterparts to be obese (18.4%) (9), and data from the Alaska Native Epidemiology Center shows comparable rates of obesity among Alaska Native children aged 2–5 (30%) (10). Similarly, Pregnancy Risk Assessment Monitoring System (PRAMS) data indicate that obesity prevalence among Alaska Native 3-year olds is higher than any other preschool group in the United States (42.2%) (11).

## Sugar-sweetened beverages and water consumption

Sugar-sweetened beverage (SSB) consumption has been previously identified as a risk factor for overweight and obesity in children and adults (12–15). Children who drink at least 1 SSB per day have 55% increased odds of becoming overweight or obese compared with children who rarely consume SSBs (16). Alaska Native adults are more than 3 times as likely (34%) as U.S. whites (12%) to consume 3 or more sugary drinks each day (8), and those living in rural locations in Alaska are significantly more likely to consume 3 or more sugary drinks each day (51%) compared with other regions of the state (8).

The reasons for these discrepancies in SSB consumption between rural versus urban residents and Alaska Native versus non-Alaska Native populations are unknown, but previous studies suggest several possibilities: (a) historical distrust of water sources (17,18); (b) a higher prevalence of lactose intolerance in Alaska Native adults, which may shape cultural expectations for milk consumption among children (19); and (c) poor access to healthy alternatives to SSBs and juices (20). The objectives of the current study were 3-fold: (a) to assess the frequency of SSB, water and milk consumption; (b) to ascertain the attitudes towards consumption of water, milk and SSB of residents of a rural, Interior Alaska Native (Athasbascan) community; and (c) to assess rural access to water, milk and SSBs. As of 2014, although there were rough, epidemiological data about consumption patterns, there were no published research describing attitudes, thedetails of daily consumption and access to beverages in Alaska Natives among the Athabascan peoples of Interior Alaska. Our research attempts to expand upon these data.

## Methods

### Population

In 2014, the authors of this study assessed the eating and drinking habits of a rural community located over 200 air miles west of Fairbanks in the interior of Alaska. The village has a population less than 300 persons, comprised of 92% American Indian/Alaska Native (50.5% female). The median age of the population in the village is approximately 26, with more than 35% under the age of 18. One of this study's authors (PDS) is employed as a physician and researcher for the U.S. government recognized non-profit, Tanana Chiefs Conference, of which the village tribe is a member. Before the initiation of this study, he made frequent visits to the village to establish wellness teams and a structure for community-based research. Village residents prioritized obesity and diabetes mellitus as important health topics to be addressed.

In the small village examined in this article, food and beverage options were limited. Local shops in the village purchase food from larger grocery and wholesale stores located in Fairbanks, and individuals occasionally order directly from these Fairbanks retailers, which is expensive, as the purchases need to be flown in on private charters. Alternatively, when village residents go to Fairbanks for medical visits, they bring food and beverages back with them on the plane. Most families rely partially on subsistence, in the form of moose meat or fish.

### Recruitment

In 2014, we visited rural Alaska to conduct focus groups and administer beverage questionnaires to assess consumption patterns. Using the village's Citizen Band (CB) radio system, the tribal administrative assistant invited village members to participate in each focus group and complete questionnaires. Flyers were also posted at local stores, the health clinic, the village post office and the village tribal centre.

Individuals who were recruited for the focus groups were also invited to fill out the beverage questionnaires. We also recruited participants at the community centre, clinic and local shops when we were introduced to village members. Any participant who completed a questionnaire, attended a focus group or completed an interview was entered into a raffle to win 1 of 3 modest door prizes valued under $60.00. We provided all focus group and questionnaire participants with 2 cookbooks produced by Alaska WIC and a sheet of children's stickers for younger participants. Fresh fruit and sandwiches were also provided at each focus group. The study was approved by Alaska Institutional Review Board (IRB), the Executive Board of Tanana Chiefs Conference, and organized under the auspices of the Tribal Wellness Teams program. All study subjects provided signed, written consent.

### Beverage questionnaire

We administered a modified version of a previously validated questionnaire, the BEVQ-15, which asks about daily and weekly beverage consumption based on consumption patterns during the last month (21). From the questionnaire, we removed 3 questions on alcohol consumption and 1 question on tea or coffee with or without artificial sweetener (no cream or sugar) consumption. These changes reduced the questionnaire to 11 items. To ensure accuracy, parents completed the questionnaire on behalf of their young children (ages 3–11), and participants 12 years and older completed their own questionnaire. We modified the questionnaire as per the Alaska IRB recommendation to remove alcohol consumption patterns, and as our initial aim was to focus on children, we removed the question about consumption of coffee and tea without added sugar or cream.

### Focus groups and interviews

We conducted 2 focus groups with community members (n=8 from first group and n=13 from second) and individual interviews with Head Start and Early Head Start program instructors as well as shop owners (n=7). Both focus groups and individual interviews used open-ended interviewing techniques with questions centred on beverage consumption in the village. Only adults were included in focus group discussions. The purpose of the focus groups was to better understand the attitudes towards SSBs, nutrition and obesity. No formal interview guide was used. Rather, the discussion during each focus group was guided by study personnel to address general themes of beverage consumption, fast food, popular items in stores, nutrition at home and at school, possible interventions and recommendations for the community.

### Shop inventory

We performed a comprehensive food and beverage inventory of all 3 shops located in the village in order to assess the availability of different beverages in the village. The authors visited the shops, assessed all items on the shelves and spoke with the storeowners to compile a list of the items being sold and the price of each item.

### Analysis

We audio recorded and transcribed all interviews and focus groups. One interviewee (a shopkeeper) declined to be recorded, so we took notes while the interview was conducted. We then performed thematic analysis to identify common beliefs and responses. We did not code the data and elected to have only 1 author perform thematic analysis to control for inter-rater reliability. We examined each of the transcripts and identified any common responses to individual questions posed by the researchers. As per our initial interests, we focused on SSB consumption habits, timing of the introduction of SSBs into children's diets, availability and restrictions on SSBs, beverage purchasing habits observed by store owners, as well as water consumption habits and attitudes towards the drinkability of village tap water.

Results from the beverage questionnaire were stratified into 3 age groups children (3–11 years), adolescents (12–17 years) and adults (≥18 years), as obesity prevalence and SSB consumption data from other studies have been stratified into these age groupings or similar ones (3,22–25) and dividing into age groups would allow us to more appropriately target future interventions. We defined total and any SSB consumption including the following beverages: sweetened juice beverages (fruitades, Tang or Kool-Aid), regular soda, sweet tea and sports/energy drinks. We did not include tea or coffee, as it was unclear whether or not sugar was added based on how the question is asked. In order to identify any possible differences in consumption patterns between young children and adolescents, we compared the results across age groups using *t*-tests for continuous variables and chi-squared tests for categorical ones. We did not compare adult consumption patterns to young children or adolescents as the dietary recommendations for intake differ for children and adults (26). Serving size was computed as a discrete variable using the beverage questionnaire. Participants had the option to select “less than 6, 8, 12, 16 or more than 20 oz.” We calculated an average serving size based on all responses for a given beverage respective of each age group. We computed the daily intake of water and milk using the average serving size and frequency of consumption reported on the beverage questionnaire for all participants that reported daily consumption of a given beverage, qualifying that this included only those participants who had greater than or equal to daily intake.

## Results

### Survey data

We obtained beverage survey data on approximately 25% of the population–25 adults (76% female), 21 adolescents (48% female) and 21 children (48% female). All adolescents and 81% of children reported drinking SSBs at least once per week in the last month, with 48% of adolescents and 29% of children reporting daily consumption, p=0.08 (Table I). Seventy-two per cent of adults reported consuming SSBs at least once per week and 20% reported daily consumption (Table III). Despite differences in body mass, mean serving size of sugary beverages was comparable between children and adolescents with a mean serving size of 286±79 mL for adolescents and 253±86 for children, p=0.27 (Table II). Adults consumed a slightly higher amount than adolescents (mean serving size=297±96 mL) (Table IV). Of all the SSBs, sweetened juice beverages such as Tang or Kool-Aid were consumed most frequently with 100% of adolescents and 76% children reporting some consumption (Table I). Furthermore, 38% of adolescents and 28% of children reported drinking these beverages daily. Adolescents were more than 3 times as likely as children to report ever drinking soda, 48% versus 15% respectively, p=0.029 (Table I). Forty-four per cent of adults reported drinking soda at least once per week, and 8% reported daily consumption (Table III). For adults and adolescents, mean serving size was highest for soda of all sugary beverages with the exception of sweetened teas (mean=302±71 mL and 315±98 mL for adults and adolescents respectively) (Table VI and Table II). Fifty-two per cent of adolescents and 29% of children reported consuming sports drinks (such as Gatorade or Powerade) or energy drinks (Rockstar, Redbull, etc.) at least once per week (Table I).

**Table I:** Child and adolescent beverage consumption pattern in an Alaskan village, 2014

Type of beverage Child (3–11), n=21 Adolescent (12–17), n=21		Never (%)	At least once per week but less than once per day (%)	At least once per day (%)	*p*^a^
Sugar-sweetened beverages					
Any sugary beverage	Child	19	52	29	0.08
	Adolescent	0	52	48	
Sweetened juice beverage/drink (Kool-Aid,	Child	24	48	28	0.06
Tang, fruitades, lemonade, punch, etc.)	Adolescent	0	62	38	
Soda/pop	Child	85	15	0	0.06
	Adolescent	53	32	16	
100% fruit juice	Child	5	86	10	0.04
	Adolescent	21	47	32	
Energy and sports drink	Child	71	29	0	0.12
	Adolescent	48	38	14	
Sweetened tea	Child	100	0	0	0.01
	Adolescent	65	30	5	
Coffee and tea (with cream and or sugar,	Child	100	0	0	0.01
includes non-dairy creamer)	Adolescent	52	24	24	
Water	Child	0	19	81	0.01
	Adolescent	25	0	75	
Milk					
Any milk	Child	9	29	62	0.26
	Adolescent	9	52	38	
Whole milk	Child	67	19	14	0.23
	Adolescent	40	35	25	
Low-fat (2%) milk	Child	24	19	57	0.04
	Adolescent	55	25	20	
Non-fat milk	Child	90	10	0	<0.01
	Adolescent	43	29	29	

a Comparison made using chi-square test.

**Table II:** Child and adolescent mean serving size

	Total Mean±SD (mL)	Adolescents Mean±SD (mL)	Children Mean±SD (mL)	*p*^a^
Sugar-sweetened beverages				
Any sugary beverage	270±83	286±79	253±86	0.27
Sweetened juice beverage/drink	240±62	247±67	233±57	0.5
Soda/pop	286±100	315±98	197±34	0.07
100% fruit juice	258±87	278±128	246±55	0.4
Energy and sports drink	337±145	328±131	355±180	0.7
Sweetened tea	315±57	315±57	0±0	NA
Coffee and tea	287±110	287±110	0±0	NA
Water	294±127	345±153	263±101	0.07
Milk				
Any milk	263±80	278±91	250±69	0.3
Whole milk	274±80	289±100	253±45	0.4
Low-fat (2%) milk	253±80	262±96	248±75	0.7
Non-fat milk	268±89	274±96	237±0	0.6

a Comparison made using *t*-test.

**Table III:** Adult beverage consumption patterns, n=25

Type of beverage Adult, n=25	Never (%)	At least once per week but less than once per day (%)	At least once per day (%)
Any sugary beverage	28	52	20
Sweetened juice beverage/drink	67	25	8
Soda/pop	56	36	8
100% fruit juice	48	40	12
Energy and sports drink	92	8	0
Water	0	21	79
Sweetened tea	74	22	4
Coffee and tea (with cream and/or sugar, includes non-dairy creamer)	12	28	60

Beverage questionnaire data indicated low levels of water consumption among residents of the village with 25% of adolescents reporting never drinking water in the past week (Table I). A significant percentage of adults (21%), adolescents (25%) and children (19%) reported not drinking water on a daily basis (Tables I and III). Forty-eight per cent of adults, 43% of adolescents and 57% of children reported drinking water at least 3 times per day. For those that reported daily consumption, mean total daily intake was 725±395 mL for children, 853±440 mL for adolescents and 890±483 mL for adults.

**Table IV:** Adult mean serving size

	Mean serving size Mean±SD (mL)
Sugary beverage (any)	297±96
Sweetened juice beverage/drink	237±55
Soda	302±71
100% fruit juice	231±49
Energy and sports drink	237±0
Water	339±139
Sweetened tea	338±143
Coffee and tea	336±143

Because of participation in WIC and the long-term storage potential of powdered milk, access to milk in this rural village was not an issue and there were no concerns about shortages. However, the questionnaire responses suggested that not all children had daily consumption with only 62% of children and 38% of adolescents reporting daily consumption of any type of milk (Table I). Mean total daily milk intake among those reporting daily consumption was 250±69 mL in children and 278±91 mL in adolescents.

### Focus group and interview data

Focus group and interview responses indicated that while many young children frequently drank Tang, Kool-Aid and even sodas, many people in the village recognized that SSB consumption was unhealthy. Nonetheless, Head Start instructors noted Kool-Aid or Tang consumption was quite common among children in the Head Start program and “pretty much the only thing [the kids drank].” Most children drank these beverages at home as federal guidelines prohibit consumption of these beverages in the Head Start or Early Head Start programs. In focus groups, parents reported trying to limit children's intake or replacing Tang or Kool-Aid with 100% juice. Study participants also indicated that SSB consumption begins early, with children as young as 1 year of age drinking SSBs in the village, and that they received no instruction in nutritional practices from healthcare providers. Young children regularly visit village shops without a guardian and are allowed to purchase soda and all SSBs.

**Shop owner –** the little ones****, if they are by themselves they buy pop [soda]. If they are with their parents, they buy Gatorade. Little kids that can barely look over the counter will buy pop.

Shop owners and consumers reported that soda was usually the first product to disappear from shelves. It was not unusual for the entire village to run out of soda.

**Interviewer –** And does the store here ever run out of things? Like shortages?

**Female focus group participant** – Oh yeah.

**Interviewer –** What things go first?

**Multiple focus group participants** (in unison) – soda pop.

In individual interviews, 2 of the 3 shop owners indicated that people in the village would not come into the store if no soda were available and that soda is responsible for generating human traffic into the stores. The owner of this shop reported that people rarely purchased 100% fruit juices unless they bought them through the Women, Infants and Children's Program. Focus group participants indicated that adolescents were buying energy drinks such as Rockstar, Redbull or Monster.

Focus group and interview participants suggested that efforts to curb SSB consumption worked well in the federally mandated Head Start and Early Head Start program, but were ineffective at maintaining low levels of SSB consumption when children were away from the programs. Similarly, research participants opined that a soda ban in schools prevented children from consuming soda in schools, but that the lack of regulations concerning children purchasing sodas in the shops undermined these efforts. Head Start teachers reported educating children in the program on nutrition, specifically soda and SSBs, but that nutritional education was not provided to all community members. At the time of the investigation, there were no initiatives in place to reduce community level soda or SSB consumption. Further, focus group responses indicated that healthcare providers failed to counsel patients on diet or nutrition and that only diabetes patients were counselled regarding nutrition.

Focus group and interview responses also indicated that village residents were not accustomed to drinking adequate amounts of water. Concern about the quality and taste of tap water was cited as reasons, and many people filtered the water to improve the taste.

**Interviewer –** Do you turn on the tap and do you drink a glass of water?

**Male focus group participant** – Myself, I use two Brita filters. I put it through one and then I run it through another one.

**Interviewer –** What's the water quality like here? When you get it out of the tap do you feel comfortable or do you feel like you need to filter it?

**Female interviewee –** I drink it … before maybe like when I was pregnant with **** – 9 years ago – it was … orange … it was almost like 3rd world country bad looking. I'm sure it was okay to drink but it didn't [look okay] … I think it was just the rust in the pipes or something.

### Inventory of stores

SSBs were sold in every store in the village. Kool-Aid or Tang were priced 75% cheaper per serving than healthier beverages such as bottled water or milk. Sixteen different varieties of sodas were sold between the 3 shops: 7-Up, Sierra Mist, Coke, Pepsi, Diet Pepsi, Wild Cherry Pepsi, Shasta Cola, Shasta Orange, Orange Crush, Strawberry Crush, Sunkist Grape, Dr. Pepper, Mtn Dew, Mtn Dew Kickstart, Mug Cream Soda and Mug Root Beer. Each shop offered between 7 and 8 different soda varieties, and there was no difference in cost based on the type of soda within a given store. Soda prices varied between the 3 stores, with the smallest store selling 12 oz. cans for $1.25, the middle-sized store charging $1.35 per can and the largest store charging $1.50 for a 12 oz. can and $3.50 for a 20 oz. bottle. Only the largest store in the village sold 100% juice, and prices ranged from $2.00 for a 10 oz. bottle to $20.00 for a 64 oz. bottle of apple juice, nearly double the price of a can of soda or half the price of a bottle of water. Bottled water was available for purchase at 2 of the village's 3 shops, but was comparably priced with soda, ranging from $1.50 for a 16.9 oz. bottle to $3.50 for a 23.7 oz. bottle compared to soda for $1.25–1.50 per 12 oz. can or juice for $2.00 per 10–11.5 oz. bottle.

## Discussion

All rural Alaska Native participants surveyed were more likely to report ever consuming SSBs than U.S. children, adolescents and adults from national survey data (2005 and 2008), with 100% of children and adolescents and 72% of adults in the village reporting consumption compared with 66% of U.S. children, 77% of U.S. adolescents and 61% of U.S. adults (22). Adolescents in the village were even more likely than Alaska high school students to report daily consumption of SSBs (48% vs. 42%) (8). We found very high rates of sweetened juice beverage consumption such as Tang, Kool-Aid or Sunny Delight in comparison with overall U.S. consumption patterns for all ages. Adolescents were nearly 5 times as likely to report ever consuming these beverages than their U.S. counterparts reported (100% vs. 23%). Similarly, children in the village were more than twice as likely as U.S. children to report ever consuming sweetened juice beverages (76% vs. 34%). as were adults (33% vs. 17%) (22). Additionally, children (29%) and adolescents (52%) in the village were more likely to report ever consuming sports and energy drinks than U.S. children (7%) or adolescents (12%) (22), or adolescents in a 2010 study on sports and energy drink consumption (37.9%) (27). The prevalence of sports and energy drink consumption we observed in the village is likely more comparable to more recent national consumption rates the percentage of American children consuming sports and energy drinks has been significantly increasing (28,29).

Overall we found a lower percentage of water consumption in the village compared with population-based surveys for adults and children, with a particular concern for adults and adolescents. A significantly greater number of adults reported no daily consumption of water (21%) than in the population-based Food Attitudes and Behavior Survey (7%) (30). Adults in the village drank less water per day (890±483 mL) than U.S. adults (1,138 mL) (31). While the percentage of village adolescents reporting daily consumption (75%) was comparable to that reported for U.S. high schools students in 2010 (72%), we had a high percentage of adolescents that reported never drinking water (25%) (32). It is possible that we found issues of low water consumption more problematic among adults and adolescents as adults remember the historical issues with water problems in the village in contrast with younger children.

Access to running water has been a challenge for many years in much of rural Alaska. In 1994, only 37% of households had access to running water (33). Between 1996 and 2010, the Environmental Protection Agency (EPA) funded projects to increase water availability in rural Alaskan villages, and, by 2010, 92% of homes had access to clean drinking water (34). This was still, however, well below the national average of 99% (34). The percentage of homes in rural Alaskan villages that had in-home piped water service was much lower, with only 73% of homes reporting in-home water service as of April 2004 (35). There are currently over 6,000 year-round occupied homes in rural Alaska that do not have running water or flush toilets (36). Further, as of 2000, one third of rural Alaska residents lacked piped water and had to obtain water from community-based water points and bring the water home in 5-gallon plastic containers. Some families have to travel long distances, even crossing rivers to obtain water from these centralized locations (35). According to the Environmental Protection Agency, the lack of safe water service poses an “extreme public health challenge” (37).

Distrust of the local water supply has also been seen in other Northern Native communities, such as First Nation communities in Canada, that have had poor historical access to water, leading residents to express health concerns associated with tap water consumption (17). The limited water availability and the historical distrust of the water supply has resulted in consumption of other, bottled or packaged beverages (18).

We also found children in the village consumed less milk per day (250±91 mL) compared with 2–4-year-old WIC participants in 2005–2010 (332 mL) (38) or U.S. children (295 mL), well below the USDA recommendations of 2 cups (473 mL) per day (39,40). In particular, adequate milk consumption is necessary for children for both calcium and vitamin D intake and has been shown to protect against obesity (41). One possible explanation for the low milk consumption may be that milk is not the best source of calcium in this population since some Alaska Native tribes have a high prevalence of lactose intolerance (19). They may have adequate calcium intake secondary to other sources in their diets; however, we did not assess calcium intake from other foods such as cheese, yogurts, ice cream or other non-dairy sources.

### Future interventions

#### Health education

Our focus groups suggest the need for health professionals to provide education on nutrition. Similarly, other studies suggest that only 43% of Alaskan adults reported ever receiving advice about their eating habits from a healthcare provider (8). While most participants in the study did recognize that soda could cause excessive weight gain and did not provide vitamins or minerals, many did not realize that other drinks such as 100% juice and juice drinks could contribute to excessive caloric intake. Ensuring patients in rural Alaska receive adequate nutritional counselling could improve patient's nutritional habits (42).

Peer-led nutritional counselling could be a viable option to decrease obesity and increase healthy living knowledge in this resource-limited environment. This approach would tie in well with the region's reliance on health aides, members of the community who serve as healthcare practitioners at the local clinics, with telemedicine support from Fairbanks, but without any formal health professional training. A First Nations community in Manitoba, Canada, had success with a peer-led intervention facilitated by high school mentors. The intervention resulted in decreased BMI and waist circumference compared with controls following the 5-month intervention. The program consisted of a 90-minute after-school program that provided a “holistic approach to physical activity, nutrition and education programming” that built upon the “strengths of the Aboriginal youths as they assume leadership roles in the community” (43). Nutritional education interventions have also had success in reducing soda consumption among Alaska Native teens. Soft drink consumption decreased by 10% following a 2-year intervention in participating Yupik villages, resulting in an 18% decrease in soda consumption in intervention villages compared with a 20% increase in control villages (44).

### School-based interventions

School-based interventions that focus on bringing nutritional education from the schools into the home and community could be another option for this region, given the high percentage of SSB consumption and low water and milk consumption, particularly in adolescence (45). Bright Start is a school-based intervention in American Indian children that increased physical activity at school, promoted healthy eating and included family focused interventions to improve the home food environment. The intervention resulted in an overall decreased prevalence of overweight among students in the intervention group compared with controls, as well as decreased SSB consumption (46). While many schools in rural Alaska limit SSB consumption, this type of comprehensive intervention that includes the home and community environment could bridge the intervention from the schools to communities.

Similarly, the *Sodabriety Challenge*, a 30-day intervention with rural Appalachian high school students, challenged students to consume only unsweetened beverages such as water, unsweetened tea or diet soda. During the intervention, participants were also given facts about the benefits of limiting sugar-sweetened beverages on a daily basis. The intervention resulted in decreased total SSB consumption at home and school both immediately following the intervention and during follow-up 30 days later. Water consumption also increased following the intervention (25). Another successful SSB initiative, *Drop the Pop*, in the Nunuvut, Northwest and Yukon territories in Canada was designed to increase awareness of the effects of SSB consumption on health by promoting healthy food days, providing instruction on how to prepare healthy drinks, meals and snacks or implementing a nutritional curriculum into the science curriculum at the school. Seventy-six per cent of schools that participated in *Drop the* Pop indicated that children were more likely to bring healthy beverages to school, and 69.8% of parents reported that the children consumed less SSBs both at school and at home following the initiative (47).

### Structural interventions

Interventions, which change the structure of beverage availability in the schools, homes or products available in stores, may have a longer lifespan than the duration of the intervention. A school-based intervention in Germany, where water fountains were installed in schools and teachers gave classroom lessons to promote water consumption, increased water consumption among students. Further, the school participating in the intervention had lower rates of obesity compared with the control school following the intervention (48). Similarly, a home-based intervention of U.S. adolescents in which participants received weekly deliveries of non-caloric beverages (e.g. bottled water, diet beverages including soft drinks, iced teas, lemonades and punches selected by participants) for 25 weeks saw greatly reduced SSB consumption among participants in the intervention group compared with the control group (23).

Finally, an intervention focusing on products available for sale in stores could be a viable model. A 14-month intervention in the Navajo Nation that sought to increase the availability of healthy foods by implementing an intervention within supermarkets, convenience stores and trading posts and to promote these foods both at the time of purchase and through community media showed promising results. During each intervention, study personnel demonstrated healthy cooking techniques, allowed residents to taste-test healthy food options, gave away promotional items and responded to questions. Greater exposure to the intervention was associated with reduced BMI and increased healthy food intentions 15–20 months following the intervention (49). Implementing an initiative to improve the nutritional habits in the village is the next step in addressing the obesity problem.

### Research strengths and limitations

This village-based study is one of the first of its kind performed in a rural, Alaska Native village (50). We used a multi-prong approach utilizing 3 forms of research in order to paint a detailed picture of an Athabascan village in Interior Alaska, elucidating potential historical, structural and attitudinal reasons for higher SSB consumption and lower milk and water consumption patterns. These data could be a starting point for multi-village observational studies in rural Alaska or for interventional work in the Athabascan Interior.

The research reported in this article has several limitations. First, the soda consumption levels obtained from the questionnaire were much lower than we predicted based on focus group and interview responses, which suggested that soda was commonly consumed at even higher rates. We believe the rates were likely underestimated in the questionnaire data, as the beverage questionnaire referred to soda as “soft drinks” and soda is better understood as “pop” in rural Alaska. Also, studies relying on recalls are limited by the vagueness of memory and estimation but we attempted to compensate for this limitation by including the focus groups to allow for qualitative study. Finally, the study took place in a single village with a relatively homogeneous Athabascan population. Other Alaska Native ethnic groups and Athabascan villages may differ substantially.

## Conclusion

Alaska Native adults, adolescents and children living in the rural interior of Alaska consume high amounts of sugar-sweetened beverages including energy drinks and insufficient amounts of water. Of all sugar-sweetened beverages, sweetened juice beverages such as Tang or Kool-Aid are consumed most frequently. Community-driven interventions targeting modified beverage consumption are needed.

## References

[1] Kuczmarski RJ, Flegal KM, Campbell SM, Johnson CL (1994). Increasing prevalence of overweight among US adults. The national health and nutrition examination surveys. 1960 to 1991. JAMA J Am Med Assoc.

[2] (1997). Overweight and obesity in the United States: prevalence and trends, 1960–1994.

[3] Ogden CL, Carroll MD, Kit BK, Flegal KM (2014). Prevalence of childhood and adult obesity in the United States, 2011–2012. JAMA.

[4] Whitaker RC, Wright JA, Pepe MS, Seidel KD, Dietz WH (1997). Predicting obesity in young adulthood from childhood and parental obesity. N Engl J Med.

[5] Pan L, Blanck HM, Sherry B, Dalenius K, Grummer-Strawn LM (2012). Trends in the prevalence of extreme obesity among us preschool-aged children living in low-income families, 1998–2010. JAMA.

[6] Hutchinson RN, Shin S (2014). Systematic review of health disparities for cardiovascular diseases and associated factors among American Indian and Alaska Native populations. PLoS One.

[7] Indian Health Service (2011). “Healthy Weight for Life: A Vision for Healthy Weight Across the Lifespan of American Indians and Alaska Natives, Actions for Health Care Teams and Leaders”.

[8] Alaska Department of Health and Social Services (2014). Alaska Obesity Facts Report — 2014.

[9] Anderson SE, Whitaker RC (2009). Prevalence of obesity among us preschool children in different racial and ethnic groups. Arch Pediatr Adolesc Med.

[10] Fenaughty AM, Fink K, Peck D, Utermohle CJ (2009). Childhood obesity in Alaska.

[11] Wojcicki JM, Young MB, Perham-Hester KA, de Schweinitz P, Gessner BD (2015). Risk factors for obesity at age 3 in Alaskan children, including the role of beverage consumption: results from Alaska PRAMS 2005–2006 and Its three-year follow-up survey, CUBS, 2008–2009. PLoS One.

[12] Ludwig DS, Peterson KE, Gortmaker SL (2001). Relation between consumption of sugar-sweetened drinks and childhood obesity: a prospective, observational analysis. Lancet.

[13] Wojcicki JM, Heyman MB (2012). Reducing childhood obesity by eliminating 100% fruit juice. Am J Public Health.

[14] De Ruyter JC, Olthof MR, Seidell JC, Katan MB (2012). A trial of sugar-free or sugar-sweetened beverages and body weight in children. N Engl J Med.

[15] LaRowe TL, Adams AK, Jobe JB, Cronin KA, Vannatter SM, Prince RJ (2010). Dietary intakes and physical activity among preschool-aged children living in rural American Indian communities before a family-based healthy lifestyle intervention. J Am Diet Assoc.

[16] Te Morenga L, Mallard S, Mann J (2012). Dietary sugars and body weight: systematic review and meta-analyses of randomised controlled trials and cohort studies. BMJ.

[17] Dupont D, Waldner C, Bharadwaj L, Plummer R, Carter B, Cave K (2014). Drinking water management: health risk perceptions and choices in first nations and non-first nations communities in Canada. Int J Environ Res Public Health.

[18] Onufrak SJ, Park S, Sharkey JR, Sherry B (2014). The relationship of perceptions of tap water safety with intake of sugar-sweetened beverages and plain water among US adults. Public Health Nutr.

[19] Suchy F, Brannon P, Carpenter T, Fernandez J, Gilsanz V, Gould J (2010). NIH consensus development conference statement: lactose intolerance and health. NIH Consens State Sci Statements.

[20] Fenaughty A, Fink K, Peck D, Wells R, Utermohle C, Peterson E (2009). The burden of overweight and obesity in Alaska, summary report. [Internet].

[21] Hedrick VE, Savla J, Comber DL, Flack KD, Estabrooks PA, Nsiah-Kumi PA (2012). Development of a brief questionnaire to assess habitual beverage intake (BEVQ–15): sugar-sweetened beverages and total beverage energy intake. J Acad Nutr Diet.

[22] Han E, Powell LM (2013). Consumption patterns of sugar sweetened beverages in the United States. J Acad Nutr Diet.

[23] Ebbeling CB, Feldman HA, Osganian SK, Chomitz VR, Ellenbogen SJ, Ludwig DS (2006). Effects of decreasing sugar-sweetened beverage consumption on body weight in adolescents: a randomized, controlled pilot study. Pediatrics.

[24] Mathias KC, Slining MM, Popkin BM (2013). Foods and beverages associated with higher intake of sugar-sweetened beverages. Am J Prev Med.

[25] Smith LH, Holloman C (2014). “Piloting “sodabriety”: a school-based intervention to impact sugar-sweetened beverage consumption in rural Appalachian high schools. J Sch Health.

[26] Dietary Reference Intakes (DRIs): recommended dietary allowances and adequate intakes, total water and macronutrients [Internet].

[27] Larson N, DeWolfe J, Story M, Neumark-Sztainer D (2014). Adolescent consumption of sports and energy drinks: linkages to higher physical activity, unhealthy beverage patterns, cigarette smoking, and screen media use. J Nutr Educ Behav.

[28] Story M, Klein L (2012). Consumption of sports and energy drinks by children and adolescents [Internet]. http://healthyeatingresearch.org/wp-content/uploads/2013/12/HER-Sports-Drinks-Research-Review-6-2012.pdf.

[29] Lasater G, Piernas C, Popkin BM (2011). Beverage patterns and trends among school-aged children in the US, 1989–2008. Nutr J.

[30] Goodman AB, Blanck HM, Sherry B, Park S, Nebeling L, Yaroch AL (2013). Behaviors and attitudes associated with low drinking water intake among US Adults, food attitudes and behaviors survey, 2007. Prev Chronic Dis.

[31] Drewnowski A, Rehm CD, Constant F (2013). Water and beverage consumption among adults in the United States: cross-sectional study using data from NHANES 2005–2010. BMC Public Health.

[32] Brener N, Merlo C, Eaton D, Kann L, Park S, Blanck HM (2011). Beverage consumption among high school students – United States, 2010 [Internet]. Center for Disease Control and Prevention.

[33] Magee G Village Safe Water Program Brief [Internet].

[34] (2012). Alaska Native villages annual report [Internet].

[35] Hennessy TW, Ritter T, Holman RC, Bruden DL, Yorita KL, Bulkow L (2008). The relationship between in-home water service and the risk of respiratory tract, skin, and gastrointestinal tract infections among rural Alaska natives. Am J Public Health.

[36] Alaska department of environmental conservation: division of water [Internet] (2013). Frequently Asked Questions.

[37] Alaska Native villages program annual report 2013 [Internet] (2013). Environmental Protection Agency.

[38] Watowicz RP, Taylor CA (2014). A comparison of beverage intakes in US children based on WIC participation and eligibility. J Nutr Educ Behav.

[39] Sebastian RS, Goldman JD, Wilkinson Enns C, LaComb RP (2010). Fluid milk consumption in the United States: what we eat in America, NHANES 2005–2006 [Internet]. *Food Surveys Research Group*.

[40] How much food from the dairy group is needed daily? [Internet] U.S. Department of Agriculture. [cited 2015 Mar 23].

[41] Song Q, Sergeev IN (2012). Calcium and vitamin D in obesity. Nutr Res Rev.

[42] Information NC for B, Pike USNL of M 8600 R, MD B, USA 20894 Behavioral Counseling to Promote Physical Activity and A Healthful Diet to Prevent Cardiovascular Disease in Adults – NCBI Bookshelf [Internet].

[43] Eskicioglu P, Halas J, Sénéchal M, Wood L, McKay E, Villeneuve S (2014). Peer mentoring for type 2 diabetes prevention in first nations children. Pediatrics.

[44] Thiele MC, Boushey CJ (1989). Soft drink consumption among Yup'ik Eskimo teenagers. Alaska Med.

[45] Caballero B, Clay T, Davis SM, Ethelbah B, Rock BH, Lohman T (2003). Pathways: a school-based, randomized controlled trial for the prevention of obesity in American Indian schoolchildren. Am J Clin Nutr.

[46] Story M, Hannan PJ, Fulkerson JA, Rock BH, Smyth M, Arcan C (2012). Bright start: description and main outcomes from a group-randomized obesity prevention trial in American Indian children. Obesity.

[47] Glacken JB (2011). Pan Territorial Evaluation of Drop the Pop [Internet].

[48] Muckelbauer R, Libuda L, Clausen K, Toschke AM, Reinehr T, Kersting M (2009). Promotion and provision of drinking water in schools for overweight prevention: randomized, controlled cluster trial. Pediatrics.

[49] Gittelsohn J, Kim EM, He S, Pardilla M (2013). A food store–based environmental intervention is associated with reduced BMI and improved psychosocial factors and food-related behaviors on the Navajo nation. J Nutr.

[50] Johnson JS, Nobmann ED, Asay E (2012). Factors related to fruit, vegetable and traditional food consumption which may affect health among Alaska Native People in Western Alaska. Int J Circumpolar Health [Internet].

